# Evaluating the advancements in protein language models for encoding strategies in protein function prediction: a comprehensive review

**DOI:** 10.3389/fbioe.2025.1506508

**Published:** 2025-01-21

**Authors:** Jia-Ying Chen, Jing-Fu Wang, Yue Hu, Xin-Hui Li, Yu-Rong Qian, Chao-Lin Song

**Affiliations:** ^1^ School of Software, Xinjiang University, Urumqi, China; ^2^ Key Laboratory of Software Engineering, Xinjiang University, Urumqi, China; ^3^ Key Laboratory of Signal Detection and Processing in Xinjiang Uygur Autonomous Region, Xinjiang University, Urumqi, China; ^4^ School of Computer Science and Technology, Xinjiang University, Urumqi, China

**Keywords:** protein function prediction, protein language model, deep learning, deep multi-label classification, gene ontology (GO)

## Abstract

Protein function prediction is crucial in several key areas such as bioinformatics and drug design. With the rapid progress of deep learning technology, applying protein language models has become a research focus. These models utilize the increasing amount of large-scale protein sequence data to deeply mine its intrinsic semantic information, which can effectively improve the accuracy of protein function prediction. This review comprehensively combines the current status of applying the latest protein language models in protein function prediction. It provides an exhaustive performance comparison with traditional prediction methods. Through the in-depth analysis of experimental results, the significant advantages of protein language models in enhancing the accuracy and depth of protein function prediction tasks are fully demonstrated.

## 1 Introduction

As key macromolecules in the life sciences, proteins play a cornerstone role in a variety of biological processes within the cell. Accurate characterization of protein function is of vital importance for disease research (Barabási et al., 2011; Xuan et al., 2019), drug discovery (Kissa et al., 2015; Zeng et al., 2016), and biotechnology advancement (Shehu et al., 2016). However, traditional experimental methods are not only time-consuming and labor-intensive but also inefficient (Colin et al., 2015; Cui et al., 2019; Torres et al., 2021). As of February 2024, while the UniProt database contains over 240 million protein sequences, less than 0.3% of these sequences have functionalities that have been experimentally validated and standardly annotated (uni, 2023). This huge gap between sequencing and annotation urgently calls for the development of efficient and reliable automated function prediction tools to save human resources and time costs (Radivojac et al., 2013).

Prior to the advent of the protein language model (PLM), numerous high-performance computational methods based on sequence similarity and deep learning have been proposed to address this challenge (Kulmanov et al., 2018; You et al., 2018; 2019; Li et al., 2024). Although these methods have made significant progress in function prediction, they fail to fully utilize the large amount of unannotated protein information. The amount of data on these unannotated proteins is growing, and the imbalance between the ratio of unannotated proteins to annotated proteins is widening (Kihara and Kihara, 2017). Furthermore, traditional deep learning methods rely on hand-designed feature extractors. These feature extractors cannot adequately capture the complexity and diversity of protein sequences, which limits the predictive power of the model (Aggarwal and Hasija, 2022; Bonetta and Valentino, 2020; Bernardes and Pedreira, 2013). The introduction of protein language models has skillfully overcome these long-standing problems and revolutionized the research field.

Inspired by the success of large-scale models in computer vision and natural language processing, the field of bioinformatics has also seen the rise of pre-trained protein language models. The introduction of the Transformer architecture has laid a solid foundation for the rapid growth of protein language models. Since the introduction of the Transformer architecture, researchers have begun to apply it to the processing of protein sequence data, and the ensuing growth of protein language models has been a springtime phenomenon. These large-scale protein language models, based on tens of millions to billions of protein sequences that are self-supervised and pre-trained, represent the state-of-the-art in predicting protein sequence function and fitness. By pre-training on huge datasets of unlabeled protein sequences, these models are capable of automatically extracting features from massive data and fine-tuning them on specific downstream tasks. Protein language models focus on three core tasks: protein function prediction, protein sequence generation, and protein structure prediction (Lin et al., 2023; Weissenow et al., 2022). These models play an important role in genomics, helping researchers to deeply interpret complex genomic data and reveal the subtle relationship between genes and proteins (Hu et al., 2024). In synthetic biology, protein language models help researchers design novel proteins or optimize the properties of existing proteins (He et al., 2024; Chen et al., 2024). In addition, in drug design, these models provide powerful support for the design and development of next-generation drugs by accurately predicting the structure of proteins and their interactions with small molecules (Zheng et al., 2024). Among the many tasks, protein function prediction, as the most basic and direct task, can intuitively reflect the effect of self-supervised training of protein language models. Therefore, this paper chooses to comprehensively review protein language models in the context of protein function prediction to comprehensively evaluate and compare the performance of these models on function prediction tasks and reveal their advantages.

Within the field of protein function prediction, the ESM 1b model (Rives et al., 2021) has attracted attention for its wide range of applications. The model achieves accurate prediction of protein function by analyzing the evolutionary information of protein sequences. The use of ESM 1b as a coding tool has significantly improved the accuracy of the protein function prediction task (Li et al., 2023; Yao et al., 2021). Not only ESM 1b but also many other protein language models can also outperform most of the protein function prediction methods in the CAFA Challenge. In recent years, emerging methods have commonly adopted pre-trained protein language models to extract sequence features (Wang S. et al., 2023; Pan et al., 2023; Zhang et al., 2023; Wang Z. et al., 2023; Kulmanov et al., 2024; Yuan et al., 2023). Thus, it has become an irreversible trend for protein language models to gradually replace the traditional sequence coding methods. In the current research context, the adoption of protein language models has become an inevitable choice if protein function prediction models are to remain competitive. In view of the central position of protein language modeling in function prediction, this review was born. By deeply analyzing and comparing the architectures, functions, training strategies, and datasets used in various protein language models, we aim to help researchers fully grasp and understand protein language models, and then be able to skillfully apply them. By effectively utilizing these advanced tools, researchers will be able to significantly improve the accuracy of protein function prediction tasks and promote their wide application in the biomedical field, which will ultimately contribute to the solution of cutting-edge scientific problems such as drug design and disease mechanism research.

This review is structured as follows: Section 2 reviews the development history of protein function prediction, and Section 3 introduces representative methods in the development history of protein function prediction methods, including statistically based methods, machine learning, and deep learning methods. Section 4 comprehensively combines through the various protein language models currently available for ontology prediction tasks, comparing their architectures, functions, and training datasets to compare the effectiveness of each protein language model in ontology prediction downstream tasks. Section 5 describes the protein sequence dataset and evaluation metrics. Section 6 shows the results and analysis of the fine-tuned protein language models on three datasets. Section 7 will select the human tRNA pseudouridine (38/39) synthetase protein as a case study, aiming to assess the prediction effectiveness and depth of different protein language models through specific examples. Section 8 summarizes this review, assesses the existing issues and trends in the field, and looks into the future direction of protein language modeling and protein function prediction.

## 2 A brief history of protein function prediction

In order to deeply explore and verify the specific functions of proteins and their mechanisms of action in living organisms, researchers first relied on biochemical experiments for protein function prediction. In 1875, science first revealed the biological function of hemoglobin, an achievement made possible by the use of the spectrophotometer (Ma et al., 2007; Thein, 2011). With this technique, scientists observed that hemoglobin can bind oxygen reversibly, thus recognizing its key function of transporting oxygen in vertebrate blood. Subsequently, between 1926 and 1930, research methods of crystallization and activity determination successfully revealed that enzymes, molecules with biocatalytic functions, are composed of proteins (Simoni et al., 2002; Manchester, 2004). Between the 1950s and the 1970s, protein isolation and purification techniques became increasingly sophisticated, with salting out, ion-exchange chromatography, gel-filtration chromatography, and affinity chromatography enabling proteins to be separated from complex cellular structures.

In the 1970s and 1980s, with the creation of protein sequence databases, scientists discovered that proteins with similar sequences often have similar functions. Using sequence comparison tools, researchers were able to hypothesize about the functions of unknown proteins by comparing them to proteins with known functions.Into the 1990s, it was gradually recognized that the key to a deeper understanding of protein function lay in accurately predicting its three-dimensional structure. Although the detailed mechanism of how proteins form their functional structures through the dynamic folding process is not yet fully understood, the concept of “structure determines function” has gradually become a consensus in the scientific community (Avery et al., 2022). With the advancement of computer technology, it became feasible to study protein behavior using molecular dynamics (MD) simulations in the late 1990s. Researchers began to use computational methods to predict protein functions from known protein structures in the Protein Data Bank (PDB) (Berman et al., 2000; 2003), thus promoting the formal formation and development of the field of protein function prediction.

From 2018, the remarkable achievements of protein language models in structure prediction have provided a great boost to protein function prediction. The breakthroughs in 3D structure prediction made by models such as AlphaFold and RosettaFold have made it possible to obtain a large number of protein structures from sequence data (Jumper et al., 2021; Baek et al., 2021). The structures predicted by AlphaFold have been proven to apply to protein function prediction (Ma et al., 2022; Gligorijević et al., 2021), with an accuracy of more than 92%, and an average error of 1 Å (Varadi et al., 2024), which is almost indistinguishable from the real structural information, effectively solving the difficult problem of mismatch between structure and massive sequence data in protein function prediction. This effectively solves the problem of mismatch between structure and massive sequence in protein function prediction.

**FIGURE 1:**
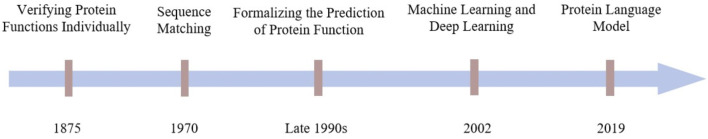
A brief history of the development of protein function prediction tasks, from statistically based methods to machine learning, deep learning to today’s protein language models.

Figure 1 illustrates the evolution of protein function prediction methods. The progression of protein function prediction has transitioned from relying on individual biochemical experiments to assess protein functions, to utilizing sequence similarity comparisons (Needleman and Wunsch, 1970), and eventually to employing computational methods based on machine learning and deep learning (Jensen et al., 2002). Each phase in this development has significantly advanced protein research and laid a robust foundation for modern, precise, and automated function prediction techniques. While each method has been instrumental in its era, they all have had their limitations. In light of this, the advent of protein language modeling is particularly pressing and significant. The emergence of protein language modeling not only represents a technological innovation but also indicates the inevitable trajectory of scientific research in harmony with the March of time (Rives et al., 2021).

## 3 Previous methods

### 3.1 Statistically based protein function prediction

The use of protein sequence homology to develop computational tools for protein function annotation was a classical early approach. This approach is based on the assumption that proteins with similar sequences usually possess similar structures and functions during evolution. Homologous proteins derive from a common ancestor and have evolved to retain key amino acids to perform similar or identical biological functions. The prediction logic is: that proteins whose functions are experimentally verified can be used as references, and proteins whose functions are unknown but whose amino acid sequences are known can be used as targets. The amino acid sequence similarity between known functional proteins and the target proteins can be calculated by using a sequence comparison tool (Pearson, 2016; Altschul et al., 1997; Remmert et al., 2012) and the similarity can be used to determine whether the target proteins have the same functions as the known functional proteins or not. It is generally believed that if the amino acid sequence similarity of two proteins exceeds 30%, they may have the same function (Chagneau et al., 2024).

In 1990, Altschul et al. (1990) developed the BLAST tool for pairwise sequence comparison, which is able to directly approximate and optimize the comparison of local similarities. BLAST first uses proteins with known functions to build a search database, then compares the target proteins in the database, ranks the comparison results according to the level of similarity, and uses the functions of the most similar proteins to infer the function of the target protein. The invention and application of BLAST marked an important milestone in bioinformatics tools, enabling scientists to more efficiently utilize the growing amount of biological sequence data to predict protein function, making it one of the most widely used tools in bioinformatics.

Released in November 2014, DIAMOND (Buchfink et al., 2015) is a highly efficient protein sequence comparison tool that uses a dual-indexing algorithm to accelerate the comparison process, making it particularly suited to the rapid analysis of high-throughput sequencing data. The core of the algorithm lies in its high speed and sensitivity, making it excellent at handling large-scale protein sequence databases. DIAMOND rapidly retrieves and matches query sequences during the alignment phase by converting protein sequences from reference databases into a compressed index format. It also introduces the use of spacer seeds to improve performance in sequence comparison. DIAMOND is used in a wide range of applications, including genome annotation, metabolic pathway analysis, and microbial community analysis. Due to its high speed and efficiency, it has become an important tool in bioinformatics research and big data analysis.

Statistical methods based on homology play an important role in the early stages of protein function prediction. However, when the amino acid sequence similarity decreases, the reliability of the prediction results of this homology-based method decreases rapidly (Devos and Valencia, 2000; 2001). When the amino acid sequence similarity between the target protein and known functional proteins is low, it is easy to generate false propagation of functional information, leading to poor prediction results. Only when the sequence similarity reaches 60% or more, do the results of homology-based inference methods have a high degree of confidence (Cruz et al., 2017). Moreover, structurally similar proteins may also possess similar functions, and structurally similar proteins may not necessarily be similar in sequence, whereas statistically based methods can only utilize sequence information. Thus statistically based methods have significant limitations in data to ensure accuracy in the task of protein function prediction, and better methods need to be proposed to meet this challenge.

### 3.2 Machine learning-based protein function prediction

Machine Learning-based Protein Function Prediction Considering protein function prediction as a multi-label, multi-classification problem, machine learning algorithms solve this problem by constructing multi-label classification models. This type of approach usually consists of four steps: feature extraction, feature selection, training the model, and classification prediction. Feature extraction involves defining and extracting sequence features, mainly in terms of compositional features, physicochemical properties, and structural features of amino acid sequences. Common protein sequence features include the frequency, position, and order of amino acid residues, as well as the hydrophobicity, polarity, and charge of amino acids, and structural domains. Feature selection, on the other hand, involves denoising and de-redundancy of the feature set obtained in the feature extraction stage to improve the training efficiency and prediction accuracy of the model. The training model stage is based on the feature set after feature selection and uses specific machine learning algorithms to build the classification model. Commonly used machine learning methods include Genetic Algorithm, KNN (K-Nearest Neighbor), and SVM (Support Vector Machine). Classification prediction, on the other hand, inputs the features of the sequence to be tested into the model built in the training phase and uses the model to determine whether the sequence to be tested belongs to the same class as a protein sequence with a specific function.

The deepNF proposed in 2018 (Gligorijević et al., 2018) uses a multimodal deep autoencoder to extract features, which are then passed to an SVM. The SVM is one of the most commonly used algorithms in the initial attempts to use machine learning techniques for protein function prediction. GODoc is a protein function prediction method that utilizes TFPSSM(Term Frequency based on PSSM) features (Liu et al., 2020). TFPSSM is a feature vector based on the frequency of the gapped dipeptides in the position-specific scoring matrix (PSSM). They proposed three different methods TFPSSM 1NN(1-Nearest Neighbor), TFPSSM CATH(Dynamic-KNN with FunOverlap), and TFPSSM Vote (Combines Fixed-KNN, Dynamic-KNN, and Hybrid-KNN voting schemes) to improve the accuracy, and also proved that the KNN variant with a dynamic voting scheme can outperform the traditional KNN method.

PANNZER (Törönen and Holm, 2022) is another tool for predicting protein function using weighted KNN classifiers, designed for automated function prediction tasks and supporting genome-level queries. KNN methods are favored for their simplicity, ease of understanding, ease of implementation, lack of need for estimating parameters, and low retraining costs. However, KNN has some limitations, such as it is a lazy learning method, computationally intensive, and the output results are weakly interpretable. In recent years, KNN has been mainly applied in the fields of text classification, cluster analysis, predictive analysis, pattern recognition and, image processing.

Protein function prediction algorithms based on shallow machine learning are able to annotate protein functions to a certain extent, but their effectiveness is often limited by noise interference in the data. The sensitivity of these algorithms to noise makes the prediction results susceptible to the quality of the data, leading to reduced accuracy. In addition, these algorithms are highly dependent on biological prior knowledge and complex feature engineering, limiting their ability to be applied to large and diverse datasets. Shallow machine learning-based methods make it difficult to achieve a qualitative breakthrough in the accuracy and coverage of protein function prediction. With the explosive growth of protein and the improvement of computational power, applying deep learning methods in protein function prediction is more promising (Radivojac et al., 2013). It provides a new way to address the limitations of current methods.

### 3.3 Deep learning-based protein function prediction

In recent years, the successful applications of deep learning techniques in computer vision, natural language processing, structure prediction, and sentiment analysis have demonstrated their powerful feature-learning capabilities (Abramson et al., 2024; Lin et al., 2023). For better proteomics research, researchers have proposed a number of protein function annotation methods that utilize deep learning techniques to extract deep features from protein characterization and integrate multiple data.

Convolutional neural networks (CNN) were first proposed in the late 1980s and early 1990s (LeCun et al., 1989), but did not gain widespread attention until after AlexNet’s (Krizhevsky et al., 2012) breakthrough performance in the ImageNet competition in 2012. CNN locally extract features through a convolutional layer, reduce spatial dimensionality through a pooling layer, and classify or regress through a fully connected layer. DeepGOPlus proposed by Kulmanov and Hoehndorf (2020). uses convolutional neural networks to extract functional features on protein sequences for annotation, which is valuable for functional annotation of a large number of newly sequenced unknown genes in macro genomes. However, the method uses amino acid solo heat codes to represent sequences, which does not take into account the semantic information of amino acids, and the sparsity of solo heat codes may adversely affect model training.

Recurrent Neural Networks (RNN) are designed for processing sequence data such as time series analysis, language modeling, and machine translation. RNN are able to process input sequences of different lengths and capture temporal dynamics in sequences through hidden states. The GONET model (Li et al., 2020) uses RNN to extract long-range links of protein sequences based on CNN to extract local features of sequences. The conserved region features related to the tertiary structure are extracted through the attention mechanism to effectively identify the protein structure domains and modalities. Thus, the prediction performance is improved.

The Transformer model, proposed by Vaswani et al. (2017) in 2017, is entirely based on the attention mechanism, discarding the traditional loop structure and effectively capturing global dependencies by considering all elements in the sequence simultaneously through the self-attention mechanism. The TALE algorithm, proposed by Cao and Shen (2021) in 2021, applies the Transformer model to protein function prediction The global features of protein sequences are extracted by the self-attention mechanism, and the hierarchical associations between functional tags are extracted by joint sequence-functional tag embedding learning, which improves the prediction performance by combining protein sequence similarity. The DeepGOA model (Zhou G. et al., 2019) innovatively introduces a graph convolutional neural network to learn the dependencies between gene ontology terms extracts the sequence features by CNN, and finally minimizes the differences between the tags and the differences in the distribution between features for function prediction.

Although deep learning methods have made significant progress in protein function prediction, they still have obvious limitations compared to protein language models. Specifically, the feature representations of deep learning methods are too sparse to reflect the complex relationships between amino acids, are less efficient in dealing with long-range dependencies and long sequences, and require significant computational resources and time for training. In addition, deep learning models usually fail to effectively integrate prior knowledge of biology, leading to unsatisfactory performance on cross-species datasets (Yang et al., 2024; Elhaj-Abdou et al., 2021). Also, the interpretability and controllability of these models are relatively weak. In contrast, protein language models are able to efficiently utilize unlabeled data through the pre-training phase to deeply mine the rich information of biological evolution, thus demonstrating a stronger capability in dealing with large-scale and complex biological data.

Protein function prediction can be likened to a natural language processing task in the field of bioinformatics, where amino acids are regarded as the basic units of a “vocabulary”and protein sequences are the equivalent of “sentences”composed of these “vocabularies”. Sentences”are composed of these “words” (Ofer et al., 2021). Compared with the traditional natural language processing problem, the protein sequence composed of 20 amino acids is closer to the character-level natural language processing. In natural language processing, the choice of an appropriate encoding method is crucial to the performance and interpretability of the model, and this principle should not be ignored in the field of protein function prediction as well. Traditional coding methods, such as one-hot coding and bag-of-words models, often fail to effectively capture the intrinsic connections between amino acids due to the sparseness of their representations. In contrast, the adoption of protein language modeling as a coding tool can better capture long-distance dependencies in sequences and provide a deeper understanding of amino acid interactions. In addition, the positional embedding function of protein language models integrates evolutionary information, providing richer and more detailed sequence characterization for protein function prediction.

## 4 Protein language modeling approach

The emergence of protein language models solves the notable problems of previous approaches by efficiently utilizing large amounts of unlabeled protein sequence data through self-supervised learning, which can identify amino acids that have remained unchanged during the evolutionary process and are often critical for protein function. Their training data contains protein sequences across multiple species, which enables the models to learn the commonalities and differences in protein sequences across species, reflecting the changing trends during evolution and capturing evolutionary information in protein sequences. These models are based on the distributional assumption that amino acids appearing in similar contexts tend to have similar meanings (Bepler and Berger, 2021). With autoregressive formulas or masked position prediction formulas, protein language models can be trained using probability distributions of amino acids to extract deep semantic information.

In an autoregressive language model, the probability of a sequence is decomposed into the probabilities of individual tokens, and the probability of each token depends only on the tokens that precede it. The drawback of this approach is that the representations learned at each location only take into account the preceding context, which may limit their effectiveness as full contextual representations. The Masked Language Modeling (MLM) approach, on the other hand, overcomes this limitation by considering the probability distribution of the tokens at each position conditional on all other tokens. Although masked language modeling does not allow the calculation of correctly normalized probabilities for the entire sequence, this approach is more appropriate when the learned representation is the main concern.

Common protein language models employ bidirectional long short-term memory networks (BiLSTM) (Huang et al., 2015), Transformer, and their variants. BiLSTM requires less training data and computational resources. As hardware resources increased and protein sequence data continued to grow, later protein language models began to adopt deep Transformer architectures, such as BERT (Devlin et al., 2018), T5 (Raffel et al., 2020), and variants of GPT (Radford et al., 2019; Madani et al., 2020; Nijkamp et al., 2023; Ferruz et al., 2022; Shuai et al., 2021; Munsamy et al., 2022) (for generative tasks). These models are trained on a large number of protein sequences to generate so-called embeddings (values extracted from the final hidden layer of the protein language model), which not only contain local and global features of the sequences, but also efficiently utilize the implicit information in the large-scale unannotated data, and can be easily migrated to a wide variety of protein prediction tasks, including functional prediction (e.g., gene ontology, signaling, binding residues or subcellular localization) and protein structure prediction, etc.

**FIGURE 2:**
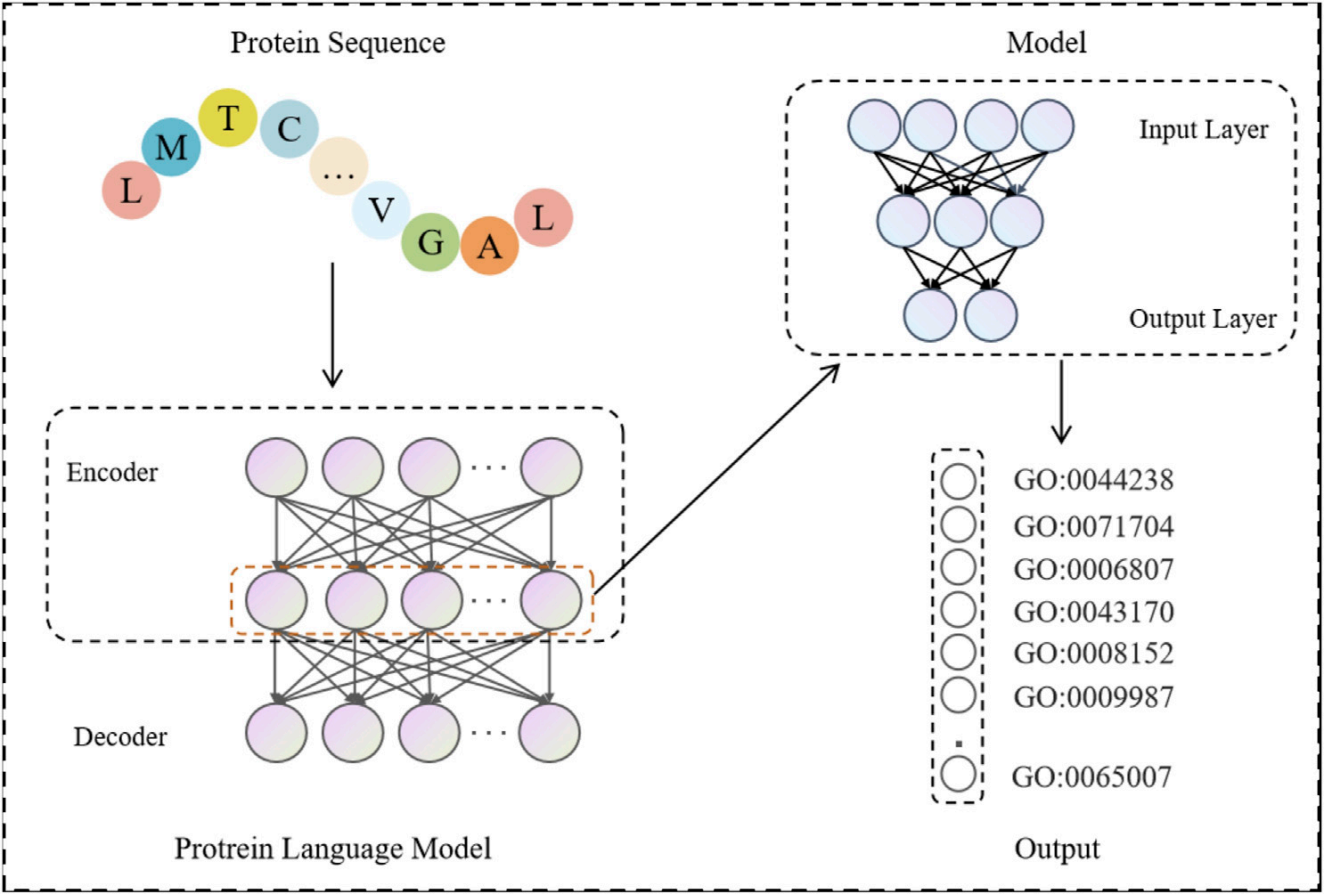
Protein sequences are fed into a pre-trained protein language model to get the output features of its encoder. These features are constructed into a feature matrix, which is then input into the model for training and testing. The final GO term probability predicted by each protein is obtained.

The process of function prediction by protein language model is shown in Figure 2. Firstly, the protein sequences are input into the pre-trained protein language model, and the features in each protein sequence are extracted using its encoder part. These features are constructed into a feature matrix, which is then fed into its own model for learning. Specifically, the feature matrix is nonlinearly transformed and features are extracted through a number of fully connected layers, which include activation functions and dropout layers between them to enhance the expressiveness of the model and prevent overfitting. Finally, the feature vectors are fed into a linear layer that maps the high-dimensional features to the final classification result space, outputting the classification results predicted by the protein function.

### 4.1 Autoregressive model

Table 1 shows the size and architecture of the encoder part of the protein language model used in this paper. SeqVec is a protein language model that employs an autoregressive model that is able to take into account previous information. It also borrows features from the BERT model, which predicts blocked words given all unblocked words. The architecture of SeqVec is based on the ELMO model using the CharCNN (Zhang et al., 2015) algorithm to obtain local features of amino acids and two layers of BiLSTM that introduce contextual information about the surrounding words. The feature vector for each amino acid is obtained by averaging the bi-directional outputs of the CharCNN and LSTM layers.

**TABLE 1:** Utilized protein language models.

Model	Base model	Dataset	Parameters (encoder)	Encoder layers	Emb.Size
ESM 1b	RoBERTa	UniRef50	650M	33	1,280
ESM2 650M	RoBERTa	UniRef50	650M	33	1,280
ESM2 3B	RoBERTa	UniRef50	3B	36	2,560
PortT5	T5	UniRef50	1.2B	24	1,024
PortBert	BERT	UniRef100	420M	30	1,024
ProstT5	T5	BFD	1.2B	24	1,024
Seqvec	ELMO	UniRef50	93M	3	1,280
Ankh Base	T5	UniRef50	450M	48	768
Ankh Large	T5	UniRef50	1.1B	48	1,536

### 4.2 Masked language modeling objective based on the BERT architecture

All models except the SeqVec model (Heinzinger et al., 2019) use a masked language modeling objective to train the model. These models take the amino acid sequence of a protein and randomly mask certain amino acids in the input sequence. The processed sequences are encoded using one-hot coding, and their representation is enhanced by positional coding and is subsequently fed into a network structure consisting of a plurality of self-attention blocks (Zhu et al., 2022). Each self-attention block contains within it multiple attention heads, linear transformation units, and feedforward neural networks. At the last attention layer of the model, the output is a probability matrix that demonstrates the model’s predicted probability distribution of amino acid species for each masked location. As the depth of the network increases, the output of each layer of the attention block forms a feature embedding that is progressively able to capture more fine-grained sequence features. These feature embeddings provide rich amino acid contextual information for subsequent protein function prediction tasks.

ProtBERT employs the BERT architecture, which is a pure encoder model without a decoder component and is particularly suited for Natural Language Understanding (NLU) tasks. ProtBERT increases the number of layers to 30 on top of the original BERT, with 420M parameters and UniRef100 protein sequence dataset to complete training. Compared to models based on convolutional neural networks and recurrent neural networks, ProtBERT uses a self-attentive mechanism to process each character in the sequence, expanding the global receptive field and enabling more effective capture of global contextual information.

ESM 1b and ESM2 (Lin et al., 2023) are protein language models based on the RoBERTa architecture (Liu et al., 2019), which improves and optimizes the traditional BERT model. RoBERTa improves performance by increasing the model size, using larger model parameters, larger batch sizes, and more training data. Unlike BERT, RoBERTa removes the Next Sentence Prediction task from BERT and employs a dynamic masking strategy that generates a new masking pattern each time a sequence is input, thus better adapting to different linguistic representations and further improving the model performance.

ESM 1b was proposed in 2020, which employs a masked language modeling objective to train the model through a self-supervised learning technique, and trains a RoBERTa model with 650M parameters and 33 layers on the UniRef50 dataset. And in 2022, ESM2 was trained using masked language modeling over millions of different natural protein evolutions with up to 15 B. During training, protein sequences are presented to the model with a portion of the residues masked, randomly aligned to different amino acids, or left unmodified. The task of the model is to predict those masked residues in a bidirectional context of all unmasked residues in the input.

Compared to traditional RNN and LSTM models, RoBERTa is able to execute concurrently, improving the computational efficiency of the model. However, static masking may result in the model not being able to adequately adapt to different masking strategies. Therefore, RoBERTa employs a dynamic masking strategy with more training data and a deeper network structure, but this also leads to longer training time and increased complexity in training and deployment.

### 4.3 Masked language modeling objective based on the T5 architecture

PortT5 (Elnaggar et al., 2021), ProstT5 (Heinzinger et al., 2023), and Ankh (Elnaggar et al., 2023) are protein language models based on the T5 (Text-to-Text Transformer) architecture. The T5 model was originally designed to deal with sequence-to-sequence problems, such as machine translation. The unique feature of T5 is that it unifies a variety of NLP tasks into a single text-to-text transformational process, by embedding the task T5 is unique in that it unifies various NLP tasks into a text-to-text transformation process by embedding the tasks into the input text to solve various NLP tasks. This design makes the T5 model highly task-adaptable and capable of being fine-tuned to accomplish many different NLP tasks.

In these models, ProstT5 further extends the initial pre-training target of ProtT5 to amino acid (AA) and 3D structure (3Di) sequences. By transforming protein structures into one-dimensional strings, conversion from sequence to structure and from structure to sequence can be achieved. However, not all protein prediction tasks directly benefit from the coupling of 3Di and AA, and may even fall short in functionally relevant tasks.

Ankh uses a T5-like architecture with a 48-layer Transformer that performs 1-g random token masking with a default probability of 20% in the input sequence and performs complete de-masking/reconstruction of the sequence. In contrast, Ankh has a larger embedding dimension, more attention heads, and more feedforward layers, which enhances the model’s representational capabilities. However, the T5 model needs to be applied and adapted with caution due to its reliance on a large amount of pre-training data and the fact that its complexity can lead to overfitting problems, especially on small datasets.

## 5 Dataset and evaluate

For the protein function prediction task, researchers can utilize two open databases, The UniProt Consortium (2023) and Protein Data Bank (PDB), to obtain protein sequence data from different species. These data can be used to train prediction models through batch downloading, data cleaning, and pre-processing. In addition, researchers can also use the CAFA dataset, which relies heavily on the Uniprot database and contains protein sequences across species. These sequences may have retained similar functions during evolution or may have undergone functional divergence. CAFA aims to assess and improve the applicability of functional prediction methods across organisms, provide standardized data to address the challenges of building computational models for protein function classification, and provide a valuable resource for evaluating and improving prediction models.

In order to standardize functional annotations, the Gene Ontology Consortium introduced Gene Ontology (GO), which classifies protein annotations into Molecular Function (MF), Biological Process (BP), and Cellular Component (CC). Molecular Function describes the role of a gene product at the molecular level, such as the catalytic activity of an enzyme or the signaling function of a protein. Biological processes involve specific biological events or pathways in which the gene product is involved, such as cell cycle regulation or immune response. Cellular components, on the other hand, are concerned with the location of the gene product within the cell, including structures such as organelles and cell membranes. As scientific knowledge continues to accumulate and be updated, the GO framework is constantly being improved to ensure its accuracy and currency as a standard for functional annotation in biological research.

**FIGURE 3:**
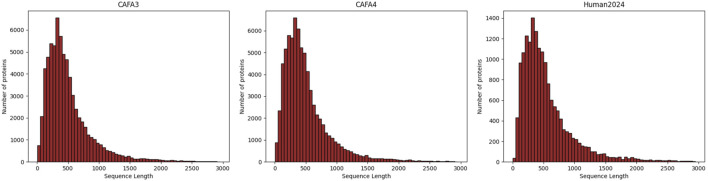
Distribution of lengths of sequences from the three datasets.

In our study, we used the human protein sequence dataset as well as the CAFA3 (Zhou N. et al., 2019) and CAFA4 datasets from DeepGOPlus. For the CAFA3 and CAFA4 datasets, we utilized the Gene Ontology (GO) data provided by the CAFA Challenge. For the human dataset, the reviewed and manually annotated human protein sequence dataset (Human2024) was collected from the SWISS-PROT (Boutet et al., 2016) database. Based on the timestamp information, we used proteins with experimental annotations obtained before 24 January 2014, as the training set, proteins with experimental annotations obtained between 24 January 2014, and 24 January 2017, as the validation set, and proteins with experimental annotations obtained between 24 January 2017, and 24 January 2024, as the test set. We used annotation information from the Gene Ontology Annotation (GOA) database (Ashburner et al., 2000; Aleksander et al., 2023) and filtered it to remove non-experimental GO annotations as well as terms not in the GO tree. Table 2 summarizes the details of the datasets used in this study. Through the statistical plots of protein lengths in the above three datasets presented in Figure 3, we can learn that most of the protein sequences are within 1,000 lengths, so we intercepted the amino acid sequences with lengths ranging from 0 to 1,000.

**TABLE 2:** Number of proteins and number of GO terms on the three sub-ontologies of the dataset.

Dataset	Ontology	Train	Valid	Test	Terms
CAFA3	MF	28,679	3,228	1,035	677
	BP	42,250	4,748	2,185	3,992
	CC	39,893	4,510	1,117	551
CAFA4	MF	25,773	7,318	3,739	725
	BP	36,423	10,445	5,236	4,507
	CC	35,972	10,284	5,129	628
Human2024	MF	6,106	2,608	676	540
	BP	6,707	792	480	2,577
	CC	8,499	1,174	1,330	398

Since the protein function prediction problem is usually transformed into a multi-label learning problem, the evaluation metrics chosen can also be based on the criteria commonly used in multi-label learning, and the following four evaluation metrics are chosen in this paper:

1. Fmax (Maximum F metric): Fmax is the maximum F metric value computed over all prediction thresholds. F metric is the harmonic mean of Precision (TP/(TP + FP)) and Recall (TP/(TP + FN)), where TP denotes true positives, number of functions of a protein that are correctly labeled, and FP denotes false positives, number of functions of a protein that should not be, but are incorrectly labeled. Where TP denotes true positives, the number of proteins whose function is correctly labeled, and FP denotes false positives, the number of proteins whose function is incorrectly labeled as a negative sample function, and FN denotes false negatives, the number of proteins whose functions are incorrectly labeled as negative sample functions.
2. AUPR (area under the precision-recall curve): AUPR is used to approximate the region under the precision-recall curve by using the trapezoidal rule, which is commonly used for the evaluation of multi-label, multi-classification tasks. A higher AUPR value indicates a better performance of the model in protein function prediction. AUPR focuses on high precision and recall, which is especially important for the precision requirement in protein function prediction.
3. AUC (area under the ROC curve): The AUC is calculated by considering all possible classification thresholds and reflects the overall classification performance of the model at all thresholds. The AUC value ranges between 0.5 and 1, where one indicates that the model classifies perfectly at all possible classification thresholds, and 0.5 indicates that the model’s classification performance is indistinguishable from a random guess. Since protein functional classes may be unbalanced, the AUC can provide a balanced assessment of the model’s performance across classes, even if some classes have fewer or more samples.
4. MCC (Matthews correlation coefficient): MCC is a metric for evaluating the performance of classifiers to effectively handle class imbalance and multi-labeled data. MCC takes into account true positives, false positives, true negatives, and false negatives across all labels. The value of MCC ranges between −1 and 1, where one indicates a perfect positive correlation, −1 indicates a perfect negative correlation, and 0 indicates no correlation. As a comprehensive metric, it is able to assess both the precision and recall of the model, ensuring a balanced consideration of the prediction results for both positive and negative samples, thus providing a more comprehensive performance assessment.

## 6 Experiments

We used PyTorch version 2.0 deep learning framework and trained the models on an NVIDIA A40 graphics card. We downloaded the following pre-trained models from GitHub and Huggingface: the ESM 1b https://huggingface.co/facebook/esm1b_t33_650M_UR50S, ESM2 650M, and ESM2 3B https://huggingface.co/facebook/esm2_t33_650M_UR50D, ProtT5 https://huggingface.co/Rostlab/prot_t5_xl_uniref50, ProstT5 https://huggingface.co/Rostlab/ProstT5, ProtBERT https://huggingface.co/Rostlab/prot_bert, Seqvec https://github.com/mheinzinger/SeqVec?tab=readme-ov-file, Ankh Base https://huggingface.co/ElnaggarLab/ankh-base, and Ankh Large https://huggingface.co/ElnaggarLab/ankh-large. During model training, we set the input dimensions of the MLP according to Table 1, and the output dimensions correspond to the number of GO terms in the sub-ontology. We used a binary cross-entropy loss function and Adam optimizer for model training, with the learning rate set to 0.0001 and a dropout ratio of 0.2 in the model. In addition, we put the batch size to 16 and the number of training rounds epoch to 100. On the validation set, we selected the model with the highest Fmax value as the final model.

In terms of model design, we adopt the architecture shown in Figure 2, where the strategy first utilizes the encoder part of each of the eight pre-trained protein language models that have been downloaded to extract features from the protein dataset. These features are constructed into a feature matrix, which is then fed into a multilayer perceptron (MLP) for processing. Specifically, the feature matrix is nonlinearly transformed and features are extracted through a number of fully connected layers, which include activation functions and dropout layers between them to enhance the expressiveness of the model and prevent overfitting. Finally, the MLP-processed feature vectors are input to a linear layer that maps the high-dimensional features to the final classification result space, outputting classification results for protein function prediction.

Through the above process, we are able to effectively utilize the advantages of deep learning models to extract deep features from protein sequences and improve the accuracy and robustness of protein function prediction through a simple MLP network structure and training strategy. This approach not only improves the generalization ability of the model but also ensures flexibility and consistency when dealing with protein sequences of different lengths.

**FIGURE 4:**
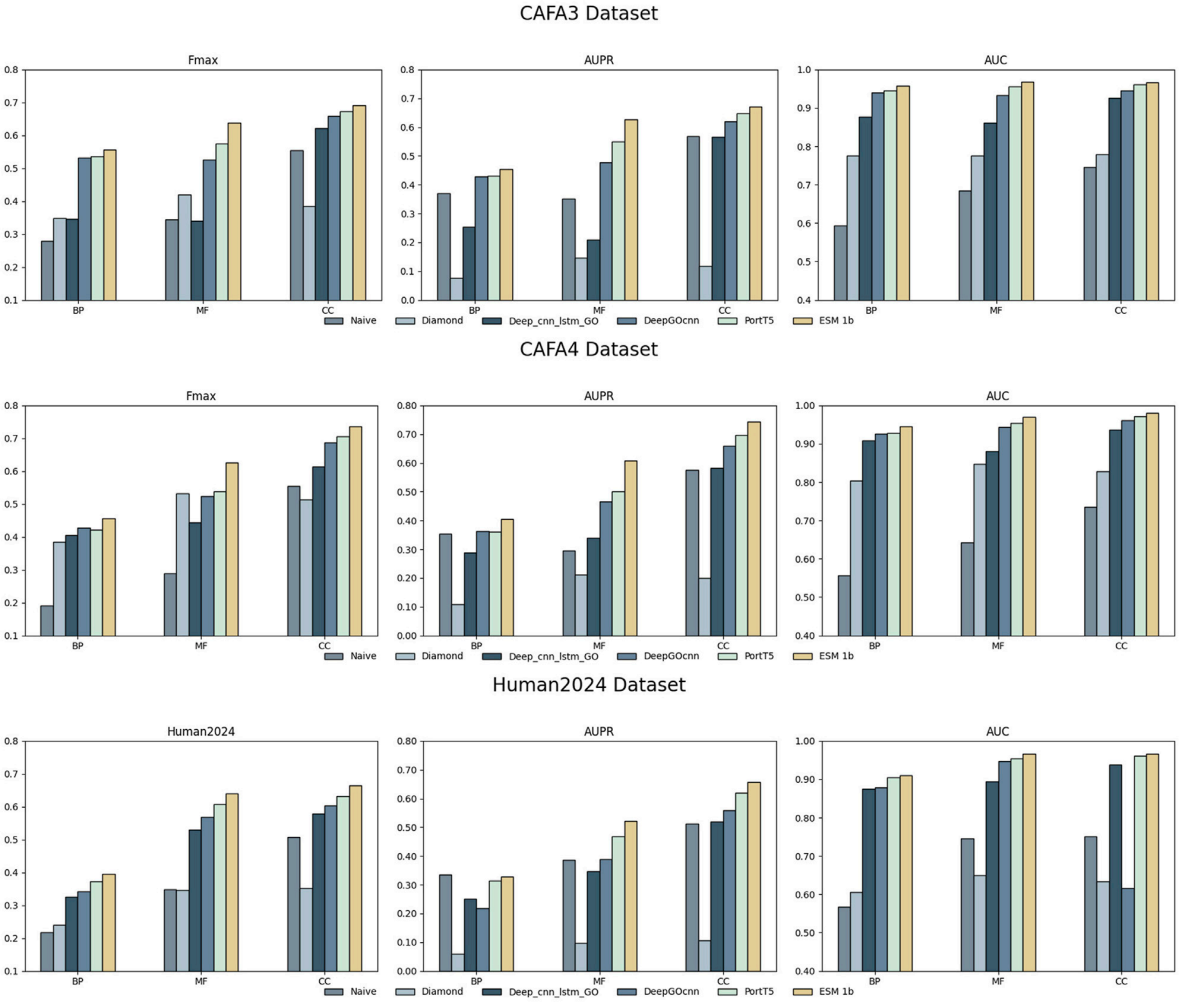
Fmax values for ESM-1b, ProtT5, and the four comparison methods on the three datasets.

In the experimental part, we used nine models, ESM 1b, ESM2 650M, ESM2 3B, ProtT5, ProstT5, ProtBERT, Seqvec, Ankh Base, and Ankh Large, to conduct comparative experiments with four methods on three datasets, Human2024, CAFA3, and CAFA4: the homology-based dual sequence comparison method Diamond, the Naive method, Deep_CNN_LSTM_GO (Elhaj-Abdou et al., 2021), and DeepGOCNN. Tables 3–5 show the Fmax, AUPR, AUC, and MCC metrics of these protein language model methods on the test set, and Figure 4 illustrates the Fmax values for the comparison experiments of ESM-1b and ProtT5 with the same four methods.

**TABLE 3:** Experimental results on the CAFA3 dataset.

Model	Fmax			AUPR			AUC			MCC		
	BP	MF	CC	BP	MF	CC	BP	MF	CC	BP	MF	CC
ESM 1b	**0.557**	**0.638**	0.691	**0.454**	**0.628**	0.671	**0.957**	0.968	0.967	**0.482**	**0.593**	0.624
ESM2 650M	0.542	0.619	0.693	0.448	0.610	0.673	0.953	0.967	0.967	0.479	0.582	**0.629**
ESM2 3B	0.549	0.622	**0.696**	0.451	0.616	**0.678**	0.955	**0.969**	**0.968**	0.478	0.575	0.628
PortT5	0.536	0.575	0.674	0.431	0.550	0.648	0.945	0.956	0.962	0.468	0.528	0.605
PortBert	0.435	0.482	0.639	0.337	0.427	0.606	0.927	0.918	0.949	0.368	0.440	0.570
ProstT5	0.521	0.557	0.671	0.404	0.514	0.643	0.940	0.947	0.954	0.442	0.504	0.601
Seqvec	0.520	0.513	0.662	0.414	0.483	0.636	0.939	0.938	0.955	0.449	0.480	0.592
Ankh Base	0.480	0.519	0.672	0.378	0.494	0.652	0.930	0.941	0.955	0.424	0.488	0.610
Ankh Large	0.441	0.504	0.667	0.362	0.471	0.647	0.927	0.935	0.954	0.400	0.476	0.604

**TABLE 4:** Experimental results on the CAFA4 dataset.

Model	Fmax			AUPR			AUC			MCC		
	BP	MF	CC	BP	MF	CC	BP	MF	CC	BP	MF	CC
ESM 1b	**0.456**	**0.626**	**0.736**	**0.404**	**0.608**	**0.743**	**0.945**	**0.970**	**0.980**	**0.418**	**0.583**	**0.671**
ESM2 650M	0.443	0.599	0.73	0.385	0.576	0.732	0.937	0.966	0.978	0.405	0.558	0.664
ESM2 3B	0.452	0.62	0.734	0.397	0.603	0.741	0.940	0.968	0.979	0.415	0.579	0.670
PortT5	0.422	0.539	0.706	0.361	0.502	0.698	0.928	0.955	0.971	0.385	0.500	0.638
PortBert	0.376	0.416	0.657	0.295	0.327	0.624	0.902	0.917	0.952	0.337	0.371	0.585
ProstT5	0.414	0.52	0.689	0.343	0.47	0.676	0.92	0.947	0.966	0.374	0.478	0.621
Seqvec	0.402	0.487	0.689	0.331	0.432	0.669	0.919	0.941	0.964	0.364	0.447	0.616
Ankh Base	0.390	0.464	0.689	0.322	0.412	0.67	0.906	0.932	0.961	0.358	0.434	0.620
Ankh Large	0.386	0.45	0.689	0.318	0.4	0.669	0.905	0.928	0.96	0.356	0.421	0.621

**TABLE 5:** Experimental results on the Human2024 dataset.

Model	Fmax			AUPR			AUC			MCC		
	BP	MF	CC	BP	MF	CC	BP	MF	CC	BP	MF	CC
ESM 1b	**0.395**	0.640	0.664	0.329	0.522	0.658	0.911	0.966	0.967	0.371	0.538	0.607
ESM2 650M	0.392	**0.670**	**0.671**	**0.332**	**0.538**	**0.668**	**0.914**	**0.970**	**0.969**	0.370	**0.566**	**0.616**
ESM3 3B	0.393	0.626	0.663	0.327	0.501	0.664	0.908	0.969	0.969	**0.373**	0.522	0.610
PortT5	0.373	0.608	0.632	0.315	0.468	0.621	0.906	0.954	0.961	0.356	0.498	0.582
PortBert	0.337	0.526	0.585	0.256	0.347	0.555	0.886	0.916	0.949	0.314	0.405	0.527
ProstT5	0.356	0.589	0.617	0.291	0.420	0.594	0.898	0.940	0.956	0.339	0.464	0.560
Seqvec	0.358	0.567	0.614	0.294	0.395	0.600	0.897	0.927	0.958	0.337	0.441	0.557
Ankh Base	0.360	0.579	0.632	0.309	0.426	0.626	0.898	0.944	0.961	0.353	0.475	0.581
Ankh Large	0.346	0.577	0.626	0.302	0.415	0.625	0.890	0.942	0.961	0.345	0.462	0.573

The Naive method, as a statistically based method, annotates proteins based on the frequency of occurrence of GO terms in the dataset. In this method, all samples in the test set are uniformly assigned with the same annotation. Diamond, as a commonly used sequence comparison tool, assigns the functions of similar proteins to the target proteins by comparing the predicted protein sequences with the training set sequences. DeepGOCNN, on the other hand, employs convolution kernels of different sizes in order to extract multiscale sequence features, and predicts the GO terms through the fully connected layer. The Deep_CNN_LSTM_GO method, on the other hand, skillfully combines the advantages of CNN and Long Short-Term Memory Networks (LSTM) to generate more reliable prediction results. For a comprehensive comparison with the protein language model, we downloaded the source code of the above four methods and implemented and evaluated them on three different datasets.

The results show that the ESM series of models achieved excellent performance on all three sub-ontologies of the CAFA3, CAFA4, and Human2024 datasets, especially on the Fmax metric. Specifically, ESM 1b achieved Fmax values of 0.456, 0.626, and 0.736 on the biological process (BP), molecular function (MF), and cellular component (CC) sub-ontologies of the CAFA4 dataset, respectively, with the best results on all three sub-ontologies, which demonstrated that the ESM 1b significantly outperforms other models in terms of the overall prediction accuracy. On the CAFA3 dataset, ESM 1b achieved the best Fmax values of 0.557 and 0.638 on the BP and MF sub-ontologies, respectively. However, on the CC sub-ontology, ESM2 3B surpassed ESM 1b with an Fmax value of 0.696 as the optimal model on this sub-ontology. In the Human2024 dataset, ESM2 650M achieves Fmax values of 0.670 and 0.671 on the MF and CC sub-ontologies, respectively, which are both optimal. On the BP sub-ontology, ESM 1b achieves a Fmax value of 0.395, which is the best result.

The ESM family of models demonstrates excellent performance on different datasets and sub-ontologies, especially in complex protein function prediction tasks. Its deep learning architecture and pre-training strategy can significantly improve prediction accuracy and coverage. The analysis shows that ESM 1b and ESM2 3B perform best on different datasets and sub-ontologies, due to their dynamic masking approach and optimization in model size, data volume, and training strategy. These results suggest that deep learning models have great potential in protein function prediction, especially when combined with large-scale data and pre-training techniques.

As can be seen in Figure 4, methods using protein language modeling are significantly better than the homology-based dual sequence comparison methods Diamond and Naive methods. frequently used methods such as ESM 1b and PortT5 outperform the convolution-based deep learning method DeepGOCNN and Deep_CNN_LSTM_GO in all the metrics. these results show that in the cross-species protein datasets CAFA3, CAFA4, and the single-species human dataset Human2024, the large language model is able to efficiently recognize GO terms for proteins, demonstrating the effectiveness of protein language models for protein function prediction tasks.

Compared to Deep_CNN_LSTM_GO, the ESM 1b model achieves a Fmax improvement of more than 10% on all sub-ontologies of both datasets. This shows that deep semantic information of sequences can be extracted using large language models. Relative to DeepGOCNN, the protein language models show less improvement on the BP and CC sub-ontologies and more improvement on the MF sub-ontology. The MF sub-ontology is usually concerned with specific molecular functions of proteins, which are more directly related to the protein’s sequence, and thus the models may be more likely to capture features related to MF. If a model architecture is better at capturing localized features, it may perform better on the MF sub-ontology. the BP and CC sub-ontologies are more concerned with the biological processes in which the protein is involved and the cellular components in which it resides, and these functions may be more relevant to the contextual environment of the protein, its interactions, and its regulatory network. These factors are difficult to infer directly from sequence data and require models with more complex structures and longer memory to capture the location and role of proteins in biological networks.

**FIGURE 5:**
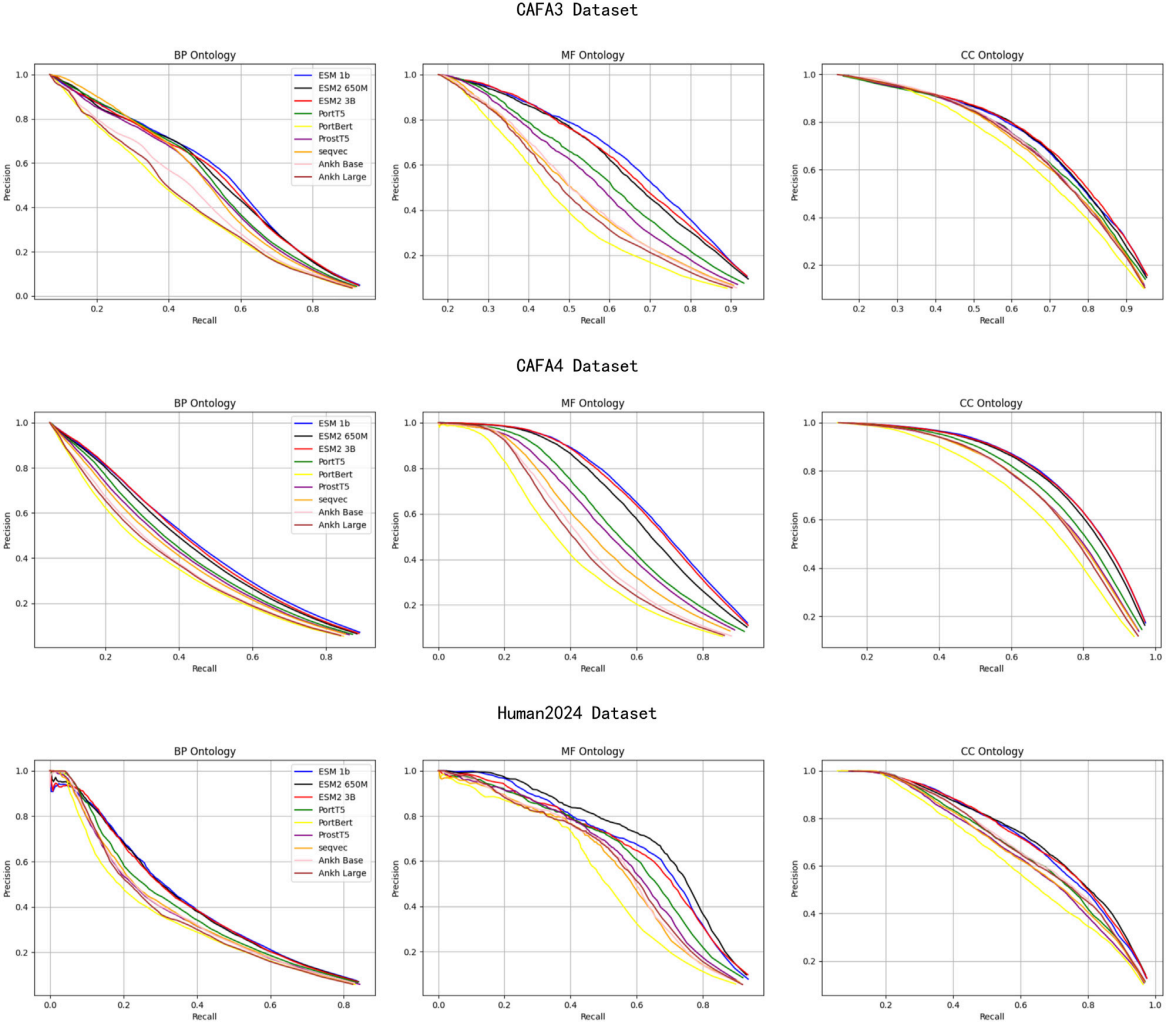
The precision-recall plots of the protein language model on the CAFA3, CAFA4 and Human2024 datasets.

Figure 5 illustrates the precision-recall (PR) plots of the protein language model on the CAFA3, CAFA4, and Human2024 datasets for evaluating the trade-off between precision and recall of the classification model at different thresholds. On all sub-ontologies of the above three datasets, the ESM family of models performs the best, while the PortBERT model has relatively low results. On the BP and CC sub-ontologies, the performance of different models is similar, but on the MF sub-ontology, the performance gap between models is more obvious. This suggests that the ESM family of models is able to better balance precision and recall when dealing with these datasets and thus performs better in the function prediction task.

These PR curve results further confirm the superiority of the ESM family of models in the protein function prediction task, especially in achieving a better balance of precision and recall when dealing with different sub-ontologies, which improves the overall performance of the models.

## 7 Case study

**FIGURE 6:**
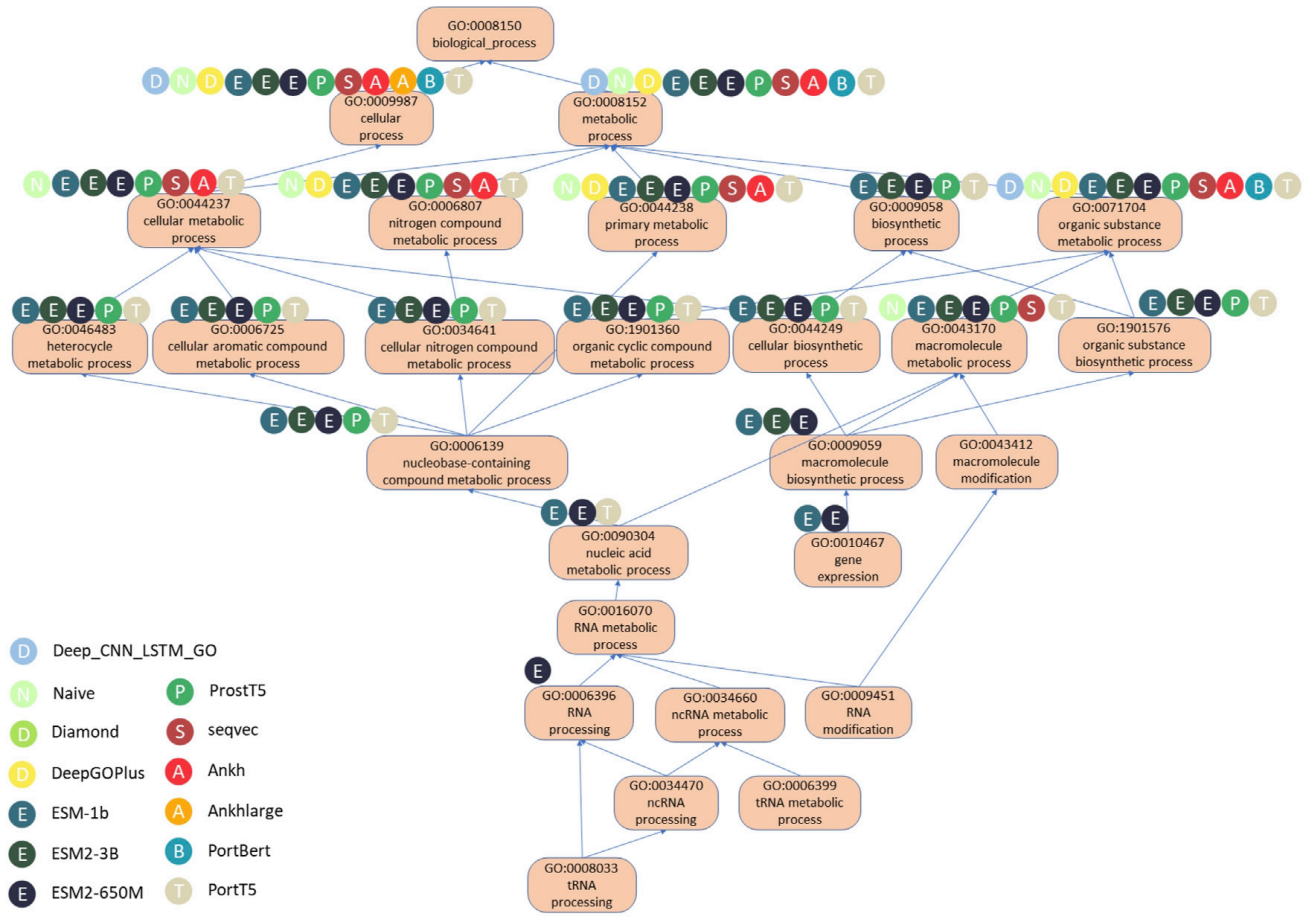
DAG diagram of correct predicted BPO terms of Q9BZE2 using different methods.

We will illustrate the differences in the performance of the various methods using the example of the protein Q9BZE2, a tRNA pseudo-uracil (38/39) synthetase that forms a pseudo-uracil at position 39 of the anticodon stem and loop of the transfer RNA. Figure 6 shows a DAG plot of the BPO terms for this protein, where the arrows represent is-a relationships, the direction they are pointing in represents the parent class, and the root term is BP. There are also methods used to correctly predict the corresponding GO terms, and Table 6 shows the GO terms correctly predicted (i.e., true positives) and the incorrectly predicted terms (i.e., false positives), as well as the F1 scores.

**TABLE 6:** Predicted GO terms for Q9BZE2 in BPO by different methods. Terms that do not appear in Labels are added*.

Method	Result	F1
Naive	GO:0009987, GO:0065007*, GO:0008152, GO:0050789*, GO:0071704, GO:0050794*, GO:0044238, GO:0044237, GO:0006807, GO:0043170	0.389
Diamond		0
DeepGOCNN	GO:0044238, GO:0071704, GO:0006807, GO:0008152, GO:0009987	0.323
Deep_cnn_lstm_GO	GO:0065007*, GO:0050789*, GO:0071704, GO:0008152, GO:0009987	0.193
ESM 1b	GO:0044238, GO:0071704, GO:0006807, GO:0043170, GO:0008152, GO:0009987, GO:0044237, GO:0006725, GO:1901360, GO:0009059, GO:0009058, GO:1901576, GO:0046483, GO:0010467, GO:0034641, GO:0044249, GO:0006139, GO:0090304	0.818
ESM2 650M	GO:0044238, GO:0071704, GO:0006807, GO:0043170, GO:1901564*, GO:0008152, GO:0009987, GO:0044237, GO:0006725, GO:1901360, GO:0009059, GO:0009058, GO:1901576, GO:0046483, GO:0010467, GO:0034641, GO:0044249, GO:0006139, GO:0090304, GO:0006396	0.826
ESM2 3B	GO:0044238, GO:0071704, GO:0006807, GO:0043170, GO:0008152, GO:0009987, GO:0044237, GO:0006725, GO:1901360, GO:0009059, GO:0009058, GO:1901576, GO:0046483, GO:0034641, GO:0044249, GO:0006139	0.762
PortT5	GO:0044238, GO:0071704, GO:0006807, GO:0043170, GO:0008152, GO:0009987, GO:0044237, GO:0006725, GO:1901360, GO:0009058, GO:1901576, GO:0046483, GO:0034641, GO:0044249, GO:0006139, GO:0090304	0.762
PortBert	GO:0071704, GO:0008152, GO:0009987	0.207
ProstT5	GO:0044238, GO:0071704, GO:0006807, GO:0043170, GO:0008152, GO:0009987, GO:0044237, GO:0006725, GO:1901360, GO:0009058, GO:1901576, GO:0046483, GO:0034641, GO:0044249, GO:0006139	0.732
Seqvec	GO:0044238, GO:0071704, GO:0006807, GO:0043170, GO:0008152, GO:0009987, GO:0044237	0.424
Ankh Base	GO:0044238, GO:0071704, GO:0006807, GO:0008152, GO:0009987, GO:0044237	0.375
Ankh Large	GO:0065007*, GO:0050789*, GO:0009987	0.069
Labels	GO:0016070, GO:0006399, GO:0008033, GO:0009059, GO:0034660, GO:0010467, GO:0009058, GO:0009987, GO:0034641, GO:0044238, GO:0044237, GO:0006725, GO:0071704, GO:0009451, GO:0046483, GO:0034470, GO:0006807, GO:0006139, GO:0043412, GO:1901576, GO:0043170, GO:0044249, GO:1901360, GO:0090304, GO:0008152, GO:0006396	

According to the data in Table 6, there are a total of 26 experimentally validated BPO terms for the Q9BZE2 protein. Of all the models evaluated, the ESM2 650M model predicted the most GO terms, with 19 of the 20 predicted terms proving to be correct and only seven terms failing to be predicted, with a Fmax score of 0.826. The ESM 1b model correctly predicted 18 GO terms, with an Fmax score of 0.816. Whereas the PortT5 and ESM2 3B models both correctly predicted 16 GO terms with Fmax scores of 0.762. It is noteworthy that only these four models successfully predicted the deep GO terms located in the lower half of the GO map, which highlights the superiority of the ESM series and PortT5 models in terms of predictive power. The PortT5 model correctly predicted 15 GO terms with a Fmax score of 0.732. These protein language models significantly outperformed the other four compared methods, confirming their ability to effectively utilize large-scale unannotated protein sequence data to deeply extract contextual information between amino acids and capture the deep semantic information of protein sequences.

It is clear from Figure 6 that the ESM family of models performs better in predicting the depth of GO terms compared to the other models. These models skillfully compute a scalar dot product of attention between the query matrix, key matrix, and value matrix in each attention header. Specifically, the model first creates a weight matrix that reveals the degree of similarity between pairs of amino acid sequences through a dot-product operation of the query and key matrices. Subsequently, the model normalizes the weight matrix using the scale parameter and the SoftMax function, a step that ensures the effectiveness and reasonableness of the allocation of attention. By multiplying the normalized weight matrix with the value matrix, the model constructs the attention matrix. As a result, the ESM2 650M model was able to accurately predict the deepest GO term, RNA processing. RNA processing is a key step in biomolecular processes that involves the conversion of preliminarily transcribed RNA into mature RNA molecules, a process that plays a decisive role in the precise regulation of gene expression.

The Diamond method based on sequence similarity encountered challenges in predicting the function of the Q9BZE2 protein due to the failure to find sequences homologous to the Q9BZE2 protein in the training set. This situation highlights the limitations of the Diamond method in dealing with uncommon or novel protein sequences. In contrast, protein language models such as ESM2 650M are able to dig deeper into the deep semantic information of protein sequences for more accurate functional prediction by virtue of their large-scale dataset utilization and advanced model architecture.

Thus, although sequence similarity-based techniques are effective in most cases, deep learning techniques, especially protein language models, demonstrate superior performance and higher prediction accuracy when dealing with complex or specific protein sequences. This case further demonstrates the advantages of protein language models in performing the task of protein function prediction, especially when confronted with challenging protein sequences. These models are able to distill more semantic information from the data, significantly improving the accuracy and robustness of the predictions.

## 8 Conclusion

The emergence of protein language models has revolutionized the field of protein function prediction. Starting from the use of the ESM 1b model in NETGO 3.0 to the wide adoption of various protein language models in many emerging protein function prediction methods today, the deep semantic information provided by these models has become an indispensable part of protein function prediction. Their tight integration has significantly improved the effectiveness of the prediction task.

In this paper, we first review the development of protein function prediction, from the initial biochemical experiments to the homology-based statistical sequence comparison methods to the application of machine learning and deep learning techniques. We sort out the key historical nodes in this field and introduce the representative methods and the problems they face in each period. Next, this paper provides a comprehensive overview of nine current protein language models that can be used for the task of gene ontology prediction, including ESM 1b, ESM2 650M, ESM2 3B, ProtT5, ProstT5, ProtBERT, Seqvec, Ankh Base, and Ankh Large. We elaborate on their architectures, functions, training strategies, and datasets, and provide an in-depth comparative analysis of them.

We have experimentally evaluated the performance of these protein language models exhaustively and compared them with other comparative methods such as traditional sequence alignment, machine learning, and deep learning. The experimental results clearly show that most of the fine-tuned protein language models significantly outperform other methods in feature encoding, which fully demonstrates the superior ability of protein language models in characterizing protein molecules. Meanwhile, the experiments also confirmed that the deep semantic information in sequences can be effectively extracted by using large-scale language models. The overall accuracy of the protein function prediction task can be significantly improved by employing protein language models.

With the continuous progress and optimization of protein language models, they gradually replace the traditional coding methods. This change has not only significantly improved the accuracy of protein function prediction, but also brought us new research perspectives and technical tools. However, despite the remarkable achievements, we still face many challenges. Among them, the size of the pre-training dataset has become a key factor constraining the development of large-scale protein language modeling (Unsal et al., 2022). Unlike the large-scale accumulation of human natural language, developing protein language models relies on advancing DNA and protein sequencing technologies. With the continuous innovation of these technologies, more and more gene and protein sequences have been identified, providing the possibility of generating large-scale and high-quality datasets. In addition, the length and complexity of protein sequences far exceed that of natural language texts, but are less diverse, which creates additional difficulty in learning and interpreting protein representations for models.

Looking ahead, the research focus will gradually shift to developing novel protein representation models capable of integrating multiple external knowledge sources. The rich connotations of proteins are closely linked to bioinformatics data such as protein-protein interactions, post-translational modifications, gene ontology, and gene and protein expression, which provide a vast scope for potential synergies between PLM and these external knowledge sources for enhancement. By supervised integration of these rich and structured resources, the capabilities of PLM will be significantly enhanced (Öztürk et al., 2019; Doğan et al., 2021). In addition, the introduction of additional resources such as physical world simulations provided by the field of molecular dynamics (MD) will greatly deepen our understanding of molecular behavior and interactions. The organic integration of PLM with MD not only complements PLM’s strengths in data processing but also strengthens its ability to analyze complex scientific phenomena, allowing for finer and more accurate interpretations (Zhang et al., 2024). In terms of coding strategies, the traditional linear positional coding can be replaced by introducing biologically relevant positional information, such as the distance matrix and contact map between sequences, to better model long-distance dependencies.
